# Occlusal Improvement, Treatment Duration, and Stability With Clear Aligners: A Systematic Review and Meta-Analysis of Peer Assessment Rating (PAR)-Based Evidence

**DOI:** 10.7759/cureus.117453

**Published:** 2026-10-04

**Authors:** Noor Y Alrefai, Mohammed A Alhazmi, Noufah M Alraithi, Anwar A Alshahrani, Leen A Suqati, Deema I Alshubaili, Raghad H Alsalhi, Randah A Almutairi, Mushari A AlQahtani, Tariq M AlOtaibi, Abdullah Koaban

**Affiliations:** 1 Orthodontics and Dentofacial Orthopaedics, Ministry of Health, Jeddah, SAU; 2 Orthodontics and Dentofacial Orthopaedics, Jazan Health Cluster, Ministry of Health, Jazan, SAU; 3 Orthodontics and Dentofacial Orthopaedics, Security Forces Hospital, Jeddah, SAU; 4 General Dentistry, King Khalid University Hospital, Abha, SAU; 5 General Dentistry, College of Dentistry, Umm Al-Qura University, Makkah, SAU; 6 General Dentistry, King Abdul Aziz University Hospital, Jeddah, SAU; 7 Dentistry, College of Dentistry, Princess Nourah bint Abdulrahman University, Riyadh, SAU; 8 General Dentistry, College of Dentistry, Majmaah University, Majmaah, SAU; 9 Orthodontics and Dentofacial Orthopaedics, First Health Cluster, Ministry of Health, Riyadh, SAU

**Keywords:** clear aligners, fixed orthodontic appliances, occlusal outcomes, peer assessment rating index, post-treatment stability

## Abstract

Clear aligner therapy is widely used as an aesthetic alternative to fixed appliances, but its ability to achieve and maintain high-quality occlusion relative to conventional braces remains uncertain when judged using comprehensive occlusal indices such as the Peer Assessment Rating (PAR) index. In this review, PAR‑based occlusal outcomes and short‑term post‑treatment stability after comprehensive clear aligner treatment are systematically reviewed and quantitatively synthesised; outcomes are compared with those of conventional fixed appliances and aligner‑only cohorts.

A systematic search was conducted in PubMed/MEDLINE, Embase, Cochrane CENTRAL, and Scopus, supplemented by Google Scholar and reference screening. Eligible studies comprised randomised controlled trials (RCTs), controlled clinical studies, and prospective cohorts that reported pre‑ and post‑treatment PAR scores (original or UK‑weighted) of adolescents or adults with permanent dentition undergoing comprehensive clear aligner therapy. Primary outcomes were total PAR reduction (absolute and percentage) and the proportion of patients classified as “improved” (≥30% PAR reduction) or “greatly improved” (≥22‑point reduction and/or final PAR = 0). Secondary outcomes included changes in individual PAR components, treatment duration, and PAR‑based stability at follow‑up. Risk of bias was evaluated using RoB 2 for RCTs and ROBINS‑I for non‑randomised studies, and the certainty of evidence was assessed with GRADE.

Seven studies (four RCTs, two retrospective comparative studies, and one prospective aligner cohort) met the inclusion criteria. Five studies contributed to the comparative meta‑analyses. Clear aligners produced large improvements in weighted PAR scores, and pooled estimates showed no statistically significant difference in overall PAR reduction compared with fixed appliances (MD −2.17 percentage points in favour of fixed appliances; 95% CI −6.74 to 2.41; I² = 73%). The proportion of patients with the outcome “great improvement” differed across the studies, with a pooled risk ratio of 0.86 (95% CI 0.69 to 1.06). In addition, nearly all participants in studies reporting this outcome achieved at least a 30% PAR reduction. Treatment duration showed no consistent difference between modalities (MD −1.48 months; 95% CI −5.45 to 2.49; I² = 96%), although heterogeneity was very high. Two non-randomised studies with follow-up PAR data reported broadly stable and low PAR scores over short- to medium-term retention; however, certainty of evidence for stability was very low due to small sample sizes, non-randomised designs, and limited comparator data.

When judged by PAR‑based global occlusal outcomes, clear aligner therapy achieves substantial occlusal improvement that appears broadly comparable to fixed appliances across selected malocclusions, without a consistent difference in the treatment duration. High‑quality RCTs with standardised PAR reporting and long‑term follow‑up are needed to define the indications for which clear aligners are considered equivalent or inferior to fixed appliances from an occlusal and stability perspective.

## Introduction and background

Clear aligner therapy, particularly the Invisalign system, is a widely used aesthetic alternative to conventional fixed appliances for managing malocclusion in adolescents and adults [1]. Recent reviews indicate that in carefully selected non‑extraction Class I cases with mild to moderate crowding, clear aligners can deliver overall alignment and occlusal improvement comparable to fixed appliances, with similar treatment times [2]. At the same time, the quality of the underlying evidence is heterogeneous, and aligner outcomes remain strongly dependent on case selection, digital planning, and biomechanical control.

Detailed clinical and in‑silico studies have begun to examine the predictability of individual tooth movements with clear aligners. Transverse expansion is feasible but often under‑expressed relative to the virtual plan, with arch‑form changes dominated by crown tipping and reduced effectiveness in maxillary canines and molars [3,4]. Three‑dimensional outcome analyses and cone‑beam computed tomography studies further demonstrate that distalization, levelling of the curve of Spee, and vertical movements such as intrusion and extrusion tend to be only partially expressed, despite the use of auxiliaries [5,6]. Furthermore, orovestibular translation tends to be more predictable in mandibular than maxillary teeth, and crown tipping is generally easier to achieve than bodily movement [7]. For premolar‑extraction cases, a recent systematic review and meta‑analysis reported that clear aligners underachieve planned anterior retraction, exhibit anchorage loss with mesial tipping of molars, and produce greater incisor tipping and extrusion than fixed appliances [8].

Studies have also examined occlusal finishing, biological response, and material behaviour. Longitudinal cohort data suggest that aligners can improve occlusal contacts and Peer Assessment Rating (PAR)‑type indices across a range of case complexities, but higher discrepancy cases are more likely to end with residual open contacts and less ideal posterior interdigitation than low‑complexity cases [9,10]. Periodontal and biomarker studies indicate that clear aligners are at least as effective as fixed appliances with respect to plaque control and gingival inflammation, supporting their use in patients with heightened periodontal risk [11,12]. In parallel, in‑vitro fatigue testing has shown that thermoplastic aligners accumulate micro‑cracks under cyclic loading and release microplastic particles, which raises new questions about long‑term biocompatibility [13]. Collectively, the available literature portrays clear aligners as effective for many crowding and Class I malocclusions, yet it highlights persistent uncertainties regarding their ability to achieve and maintain high‑quality occlusion, particularly in complex extraction cases and movements demanding precise control of sagittal, transverse, and vertical relationships [14].

Most existing research prioritises dimensional accuracy or movement‑specific outcomes and only secondarily takes into account global occlusal indices, such as the PAR, Objective Grading System, or related composite scores. Furthermore, evidence on post‑treatment stability after aligner therapy remains fragmented, with few PAR‑based follow‑up studies and limited data on relapse patterns by age or case complexity. Therefore, a review is needed to evaluate the performance of Invisalign and other clear aligner protocols based on comprehensive occlusal indices that integrate anterior alignment, anteroposterior, transverse, and vertical posterior relationships, overjet, overbite, and midline. In addition, a comparison of these outcomes against those of fixed appliances across different malocclusion severities and extraction patterns is needed.

The present systematic review and meta‑analysis concentrates on PAR‑based occlusal outcomes and short‑term stability after comprehensive clear aligner treatment, with and without direct fixed‑appliance comparators. By analyzing outcomes and pooling comparable outcomes across randomised and controlled clinical studies that report weighted PAR scores at baseline, treatment assessment points, and where available at follow‑up, this review aims to compare the performance of clear aligners and fixed appliances in global occlusal correction. It aims to identify which PAR components (anterior alignment, anteroposterior, transverse, vertical, overjet, overbite, midline) show differential performance and to explore whether treatment duration and early stability differ meaningfully between modalities. In doing so, it seeks to transform movement‑level insights from recent biomechanical and digital‑planning research into a clinically oriented, outcome‑based framework that is directly relevant to everyday treatment planning and patient counselling.

## Review

Protocol design and database search strategy

The Population, Intervention, Comparison, Outcomes, and Study design (PICOS) framework was used to determine the eligibility criteria [15]. The population (P) included adolescents aged at least 12 years and adults with permanent dentition who are undergoing comprehensive orthodontic treatment with clear aligners, with or without premolar extractions. Eligible malocclusions ranged from mild to severe and included mild-moderate Class I and Class II division 1 cases suitable for non‑extraction management as well as more complex malocclusions requiring extraction protocols, such as four first‑premolar extractions. Patients were excluded if they were in the primary or mixed dentition period, had syndromes, craniofacial anomalies, or indications for combined orthodontic-orthognathic surgical treatment, presented with active periodontal disease or extensive restorative needs at baseline, or received only interceptive, adjunctive, or otherwise non‑comprehensive orthodontic treatment that did not aim for full alignment and occlusal finishing.

The intervention (I) was comprehensive orthodontic treatment delivered with clear aligner therapy. This included commercial systems such as Invisalign, based on sequential thermoplastic trays and digital treatment planning software (for example, ClinCheck), as well as in‑house or laboratory‑fabricated clear aligner systems constructed from a staged diagnostic setup. Adjunctive procedures that formed a part of contemporary aligner protocols, such as bonded attachments, interproximal enamel reduction, inter‑arch elastics, and refinements or additional aligner series, were permitted and recorded.

Three a priori comparison strata (C) were defined. The primary comparison, which addressed the core review question, compared clear aligner therapy with conventional fixed appliances, typically labial straight‑wire edgewise brackets or equivalent systems. Eligible studies included parallel groups in which clear aligner treatment was directly compared with fixed appliances under broadly comparable baseline malocclusion severity and treatment objectives. A first secondary comparison examined variations within aligner protocols, such as remote Dental Monitoring versus conventional in‑office monitoring. In addition, single‑arm cohorts of comprehensive clear‑aligner treatment without a concurrent fixed‑appliance control were included to characterise the overall occlusal improvement and short‑term stability after treatment. Studies had to include at least one clear‑aligner treatment arm to be eligible for inclusion.

In order to be included, studies had to report occlusal outcomes (O) using the PAR index, in either its original or UK‑weighted form [16]; studies that used only other occlusal indices were not eligible for the main quantitative synthesis. The primary outcomes were defined beforehand. The total PAR scores at baseline (T0) and at the end of active treatment (T1) were required, using the original or UK‑weighted PAR index and incorporating all eight components (maxillary and mandibular anterior alignment, anteroposterior, transverse, and vertical buccal relationships, overjet, overbite, and midline). Occlusal improvement was evaluated using the following reported measures: absolute reduction in PAR, calculated as PAR at T0 minus PAR at T1; percentage reduction in PAR, calculated as (PAR at T0 − PAR at T1) / PAR at T0 × 100%; and/or proportions of patients achieving pre‑specified PAR‑based thresholds. These thresholds included an “improved” outcome, defined as at least a 30% reduction in PAR, and a “greatly improved” outcome, originally defined as a reduction of at least 22 PAR points [16]. Secondary outcomes focused on the quality and pattern of correction, treatment efficiency, and post‑treatment stability. Where reported, changes in individual PAR components, such as anterior alignment, anteroposterior, transverse and vertical relationships, overjet, overbite, and midline, were used to analyse differential patterns in the occlusal correction achieved by clear aligners compared with fixed appliances. Treatment duration was measured as the total time in months from appliance placement or delivery of the first aligner to debonding or delivery of retainers; where available, PAR reduction per month was included as an efficiency metric. Stability and relapse were evaluated using PAR scores at post‑treatment (T1) and at follow‑up (T2), with a minimum interval of six months after completion of active treatment, and the change in PAR between T1 and T2. Studies that reported only other occlusal or functional metrics, such as the ABO Objective Grading System, T‑Scan‑derived contact measures, bite force, or arch‑width changes, without PAR data were excluded.

Eligible study designs (S) included randomised controlled trials (RCTs) comparing clear aligners with fixed appliances or comparing different monitoring or management strategies within clear aligner therapy; prospective or retrospective controlled clinical studies with parallel clear‑aligner and fixed‑appliance groups that were matched as closely as possible for baseline malocclusion severity and key demographic variables; and prospective single‑cohort studies of comprehensive clear‑aligner treatment that reported pre‑, post‑, and, where available, follow‑up PAR scores used to inform estimates of effectiveness and stability. Case reports and uncontrolled case series without PAR‑based group‑level outcomes, cross‑sectional studies lacking longitudinal pre‑ and post‑treatment PAR data, and non‑clinical investigations such as in‑vitro, simulation, or purely digital‑planning studies without patient‑level PAR outcomes were not considered eligible.

Database selection and search strategy

The literature search strategy was designed to identify all studies evaluating occlusal outcomes of clear aligner therapy using the Peer Assessment Rating (PAR) index, including comparative studies against fixed appliances and aligner‑only cohorts. Electronic searches were conducted in PubMed/MEDLINE, Embase, the Cochrane Central Register of Controlled Trials (CENTRAL), and Scopus from database inception to the final search date (17 March 2026). To improve capture of in‑press and non‑indexed material, supplementary searches were performed in Google Scholar and by screening reference lists of included studies and relevant systematic reviews.

The search strategy was based on three core concepts derived from the terminology used in key PAR‑based clear‑aligner trials: (1) clear aligners and Invisalign; (2) fixed appliances/orthodontic treatment; and (3) the PAR index. For the clear‑aligner concept, free‑text synonyms comprised “Invisalign”, “clear aligner”, “clearaligner”, “aligner therap”, and “clear aligner therap”. The fixed‑appliance/orthodontic concept involved terms such as “fixed appliance”, “fixed orthodontic appliance”, “conventional brace”, “labial bracket”, and “orthodontic appliance”, capturing the comparators used in PAR‑based studies of Invisalign versus conventional fixed appliances. The outcome concept specifically sought out the PAR index using the phrases “Peer Assessment Rating”, “PAR index”, and “PAR score”. The database-specific search strategies are presented in Table 1.

**Table 1:** Database-Specific Search Strategies MEDLINE: Medical Literature Analysis and Retrieval System Online; MeSH: Medical Subject Headings

Database	Search Strings
PubMed	#1 Clear aligners ("Invisalign"[tiab] OR "clear aligner"[tiab] OR "aligner therap"[tiab]) #2 Fixed appliances / orthodontic treatment ("fixed appliance"[tiab] OR "fixed orthodontic appliance"[tiab] OR "conventional brace"[tiab] OR "orthodontic appliance"[tiab] OR "orthodontic treatment"[tiab]) #3 PAR index ("Peer Assessment Rating"[tiab] OR "PAR index"[tiab] OR "PAR score"[tiab]) #4 Combine #1 AND #3
Embase	1. 'invisalign'/exp OR invisalign:ti,ab,kw 2. ("clear aligner" OR "aligner therap"):ti,ab,kw 3. 1 OR 2 4. 'orthodontic appliance'/exp OR 'orthodontic bracket'/exp 5. ("fixed appliance" OR "fixed orthodontic appliance" OR "conventional brace"):ti,ab,kw 6. 4 OR 5 7. ("peer assessment rating" OR "PAR index" OR "PAR score"):ti,ab,kw 8. 3 AND 7 9. 3 AND 6 AND 7 10. Limit 8 OR 9 to: humans AND article AND (english language)
Cochrane Library (CENTRAL)	#1 (Invisalign OR "clear aligner" OR "aligner therap"):ti,ab,kw #2 ("Peer Assessment Rating" OR "PAR index" OR "PAR score"):ti,ab,kw #3 (orthodontic OR "fixed appliance" OR "conventional brace" OR "orthodontic appliance"):ti,ab,kw #4 #1 AND #2 #5 #1 AND #2 AND #3
Scopus	(TITLE-ABS-KEY(Invisalign) OR TITLE-ABS-KEY("clear aligner") OR TITLE-ABS-KEY("aligner therap*")) AND (TITLE-ABS-KEY("Peer Assessment Rating") OR TITLE-ABS-KEY("PAR index") OR TITLE-ABS-KEY("PAR score")) AND (TITLE-ABS-KEY(orthodontic) OR TITLE-ABS-KEY("fixed appliance") OR TITLE-ABS-KEY("conventional brace") OR TITLE-ABS-KEY("orthodontic appliance"))
Google Scholar	"clear aligner" "PAR index" orthodontic "clear aligner therapy" "Peer Assessment Rating"

Except for the Embase database, where the search was limited to human, article, and English-language results, no publication‑type, date, or study‑design filters were applied at the search stage; language was restricted to English only during screening if non‑English abstracts appeared in the results. All retrieved records were exported to a reference manager (Zotero, Corporation for Digital Scholarship, Vienna, Virginia, US), where automatic and manual procedures were used to remove duplicates. The de‑duplicated records were screened in two stages against the pre‑specified PICOS criteria: first by title and abstract, and subsequently by full text for potentially eligible reports. At both stages, particular attention was paid to the presence of at least one clear‑aligner treatment arm and the availability of PAR‑based occlusal outcomes. Apart from Embase, non‑English articles were not excluded at the search stage, but records for which neither the abstract nor the full text was available in English were excluded during screening.

Assessment of methodological quality

Risk of Bias Assessment

Risk of bias was assessed at the study level separately for RCTs and non‑randomised studies (NRS) in line with contemporary Cochrane guidance and Preferred Reporting Items for Systematic Reviews and Meta-Analyses (PRISMA) 2020 recommendations [17]. Two reviewers independently assessed all studies; disagreements were resolved by discussion and, when necessary, consultation with a third reviewer.

For RCTs, the revised Cochrane risk‑of‑bias tool (RoB 2) was used [18]. Judgements were made within the five standard domains: (1) bias arising from the randomisation process, (2) bias due to deviations from intended interventions, (3) bias due to missing outcome data, (4) bias in measurement of the outcome, and (5) bias in selection of the reported result. Each domain, and the overall risk of bias, was rated as “low risk”, “some concerns”, or “high risk” following the decision algorithms. For presentation, domain‑level RoB 2 assessments were entered into RevMan [19] to generate a traffic‑light plot for the RCTs. The ROBINS‑I tool was used for NRS (retrospective and prospective cohorts and case‑control studies), as it is designed to evaluate the risk of bias in non‑randomised studies of interventions [20]. The seven ROBINS‑I domains were assessed: (1) confounding, (2) selection of participants, (3) classification of interventions, (4) deviations from intended interventions, (5) missing data, (6) measurement of outcomes, and (7) selection of the reported result. Each domain, and the overall risk of bias, was judged as “low”, “moderate”, “serious”, “critical”, or “no information”. These ratings were imported into the robvis package to produce risk‑of‑bias traffic‑light plots and summary bar plots for the non‑randomised evidence [21].

Statistical Analysis

The Cochrane Review Manager (RevMan Web, Version 10.2.0) [19] was used to quantitatively assess the studies, and a forest plot was used to visualise the meta-analysis results. For percentage PAR reduction and treatment duration, group means, standard deviations, and analysed sample sizes were extracted directly from the primary-study tables; no standard deviations were imputed. The random effects model was chosen to account for the variability in terms of malocclusion severity, extraction status, aligner systems, retention protocols, and study design outcomes in the included studies. Heterogeneity was quantified using the I² statistic, with values above 50% considered substantial. A narrative synthesis was provided if meta-analysis was not feasible. To complement the synthesis of the outcomes, the overall certainty of evidence was evaluated using the GRADE approach [22].

Results

Database Search Yields

The database searches yielded 487 records in total (PubMed n = 124, Scopus n = 89, Embase n = 46, CENTRAL n = 20, and Google Scholar n = 208). Next, 81 records were removed prior to the screening process. Of these, 56 were duplicates and 25 were removed for other reasons; no records were excluded by automated eligibility-screening tools. Then, the titles and abstracts of the remaining 406 records were screened. Title and abstract screening excluded 201 records that clearly did not meet the inclusion criteria, leaving 205 reports that were sought for retrieval. As four reports could not be retrieved, there were 201 full-text articles for detailed eligibility assessment. Of these, 194 full-text reports were excluded because they did not include a clear-aligner treatment arm (n = 53), did not report PAR-based occlusal outcomes (n = 128), were case reports/series without group-level PAR data (n = 5), or had ineligible study designs (n = 8). Ultimately, seven studies met the inclusion criteria and were included in the qualitative synthesis, five of which contributed data to at least one comparative meta-analysis. The flow of studies through the identification, screening, eligibility, and inclusion phases is summarised in the PRISMA 2020 flow diagram shown in Figure 1.

**Figure 1:**
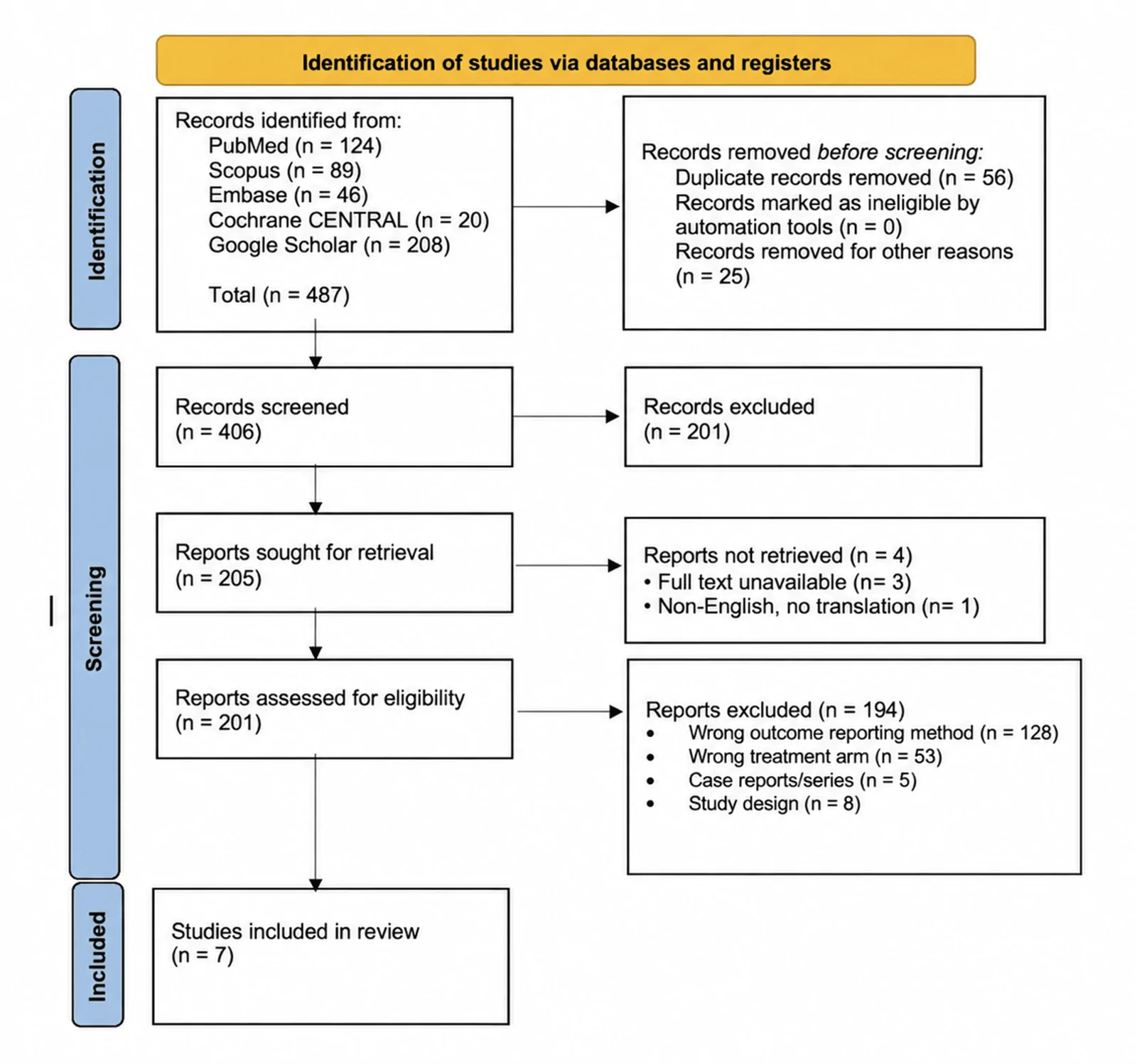
PRISMA 2020 Flow Diagram of Database Search and Article Selection Process PRISMA: Preferred Reporting Items for Systematic Reviews and Meta-Analyses

Across the seven included studies, clear aligner therapy resulted in large improvements in weighted PAR scores, and pooled estimates indicated little to no difference in overall occlusal improvement compared with conventional fixed appliances. Table 2 summarises the findings of the included studies.

**Table 2:** Summary of Characteristics and Outcomes of Included Studies CA: clear aligners; FA: fixed appliances; CAT: clear aligner therapy; RCT: randomised controlled trial; PAR: Peer Assessment Rating; DM: Dental Monitoring; CM: conventional monitoring; IPR: interproximal reduction; MBT: McLaughlin–Bennett–Trevisi prescription; NiTi: nickel–titanium; SS: stainless steel; TPA: transpalatal arch; SAMMER: stepwise activated movements by multiple enhanced re-anchorage; T0: baseline; T1: treatment assessment (12 months in the study by Alam et al. [23]; treatment completion in the other studies); T2: post-treatment follow-up; N: sample size; y: years.

Study (year)	Country/setting	Design	Comparison	Sample (N, age)	Malocclusion & extraction status	Interventions	PAR time‑points	Follow‑up	Key findings
Alam et al. (2024) [23]	Saudi Arabia; university/hospital	Prospective parallel‑group RCT	Primary CA vs. FA (12‑month interim)	100 adults (50 CA, 50 FA); 18–50 y	Mild–moderate malocclusions; good oral health; no severe Class III or surgical cases; comprehensive treatment planned for 12–18 months	Invisalign clear aligners vs. conventional metal braces; full‑arch comprehensive alignment with standard protocols	PAR at baseline (T0) and 12 months of treatment (intermediate T1); % PAR reduction and between‑group difference reported	No T2 stability; duration beyond 12 months described but PAR is only at 12 months	Adult RCT on CA vs. FA using 12‑month PAR as outcome; also reports treatment time, satisfaction, and discomfort.
Gaikwad et al. (2025) [24]	India; tertiary care dental hospital	Prospective parallel‑group RCT	Primary CA vs. FA	120 randomised (60 CA, 60 FA); 112 analysed (55 CA, 57 FA); adults 18–35 y in permanent dentition	Mild–moderate Class I/II div 1; both non‑extraction and extraction cases (18/55 CA and 20/57 FA underwent extractions); non‑syndromic, good periodontal health	Invisalign Full vs. 0.022‑slot MBT fixed appliances; full‑arch comprehensive treatment with attachments, IPR, elastics as needed	PAR at baseline (T0) and end of active treatment (T1); % PAR reduction reported	No formal post‑retention PAR; treatment duration in months and PAR/month reported	Adult RCT on mild–moderate cases; PAR reduction was 78.4% with CA and 82.1% with FA; the reported p-value is inconsistent with recalculation from the published summary data; longer duration for CA and higher satisfaction/lower pain vs. FA
Graf et al. (2021) [25]	Germany; two orthodontic centres	Prospective double‑centre cohort	Secondary comparison B – single‑arm CAT cohort	33 adult Invisalign patients selected from 98 screened; ≥18 y; mean ≈32 y	Mixed Class I/II/III; mostly crowding; full permanent dentition; non‑extraction; same retention protocol across sample	Invisalign only; average 72 aligners and ~12 attachments per case; weekly changes; some refinements; standardized mandibular multistrand bonded retainer and maxillary modified Hawley retainer	UK‑weighted PAR at baseline (T0), end of aligner treatment (T1), and ≈10‑month retention (T2); % PAR reduction and PAR categories (improved / greatly improved) reported	Mean 10.1‑month retention (≈half of active treatment); PAR‑based stability (T1–T2) analysed by components (anterior, buccal, overjet, overbite, midline)	Shows 77% mean PAR reduction with all cases improved/greatly improved and stable PAR during short‑term retention; provides data on treatment planning parameters (number of aligners, ClinCheck revisions, refinements) and their association with outcomes
Gu et al. (2017) [26]	USA; university graduate orthodontic clinic	Retrospective controlled clinical (case–control)	Primary CA vs. FA	96 patients (48 Invisalign, 48 FA) matched on baseline PAR, age ≥16 y, malocclusion	Mild–moderate Class I/II; mostly non‑extraction comprehensive cases; full permanent dentition	Invisalign system vs. various pre‑adjusted straight‑wire labial fixed appliances; standard NiTi–SS sequences	UK‑weighted PAR at T0 and T1; absolute and % PAR reduction, classification into improved (≥30%) and greatly improved (≥22 points or final PAR 0)	No T2 data; treatment duration and PAR/month efficiency reported	Matched cohort showing similar PAR correction in mild–moderate cases, with efficiency differences between Invisalign and fixed appliances
Jaber et al. (2022) [27]	Syria; university dental school	Single‑centre 2‑arm RCT	Primary CA vs. FA (complex extraction)	36 adults (18 CA, 18 FA); 18–25 y; mean ≈21 y	Class I dental/skeletal; severe crowding >6 mm; all four first‑premolar extractions; no systemic disease; permanent dentition	In‑house clear aligners (clinic‑fabricated, thermoformed over 3D‑printed models, SAMMER staging protocol) vs. conventional 0.022‑slot MBT fixed appliances with TPA/Nance/lingual arches for anchorage	UK‑weighted PAR at pre‑treatment (T0) and end of active treatment (T1); component PAR domains, absolute and % reduction, improved (≥30%) and greatly improved (≥22 points) proportions	No post‑retention PAR; stability not assessed	First RCT on in‑house aligners in four‑premolar extraction cases; shows comparable PAR improvements and similar treatment duration CA vs. FA in complex crowding
Lanteri et al. (2018) [28]	Italy; single private specialist + university link	Retrospective matched cohorts	Primary CA vs. FA (with long‑term stability)	200 patients: 100 Invisalign, 100 FA; ages 14–56 y (mean ≈ mid‑20s); 155 at follow‑up (80 Invisalign, 75 FA)	Anterior crowding (both arches); full permanent dentition; non‑extraction; matched on sex, age, Little’s index, and baseline PAR	Invisalign vs. straight‑wire MBT fixed appliances; both arches treated; same bonded canine‑to‑canine retainers in mandible and lateral‑incisor‑to‑lateral‑incisor in maxilla; aligner group also used modified last aligner at night	UK‑weighted PAR at pretreatment, post‑treatment (T1), and ≈2‑year follow‑up (T2); % improvement and improved/greatly‑improved categories reported	Minimum 2‑year post‑treatment follow‑up; PAR‑based stability and Little’s index relapse assessed	Large cohort demonstrating high PAR improvements (>80%) and comparable 2‑year occlusal stability for Invisalign and FA under a robust bonded‑retainer protocol
Marks et al. (2024) [29]	Australia; university + private practices	Retrospective secondary analysis of an RCT	Secondary comparison A – DM vs. conventional monitoring within CAT	51 CAT patients from initial RCT: 25 DM, 26 CM; age 12–54 y; majority female	Permanent dentition with ≥16 aligners planned in both arches; non‑extraction; no auxiliary appliances beyond CAT itself; relatively simple–moderate cases	All patients treated with Invisalign; randomisation to remote Dental Monitoring (DM) vs. conventional in‑office monitoring (CM) only; otherwise identical aligner protocols	UK‑weighted PAR at pretreatment and post‑treatment; absolute and % reductions; proportions “improved” (≥30 %) and “greatly improved” (≥22 points) by Richmond criteria	No T2 stability; analysis limited to pre‑/post‑treatment PAR over ≈11–12 months of CAT from parent RCT	Demonstrates significant PAR improvement in both groups with no statistically significant difference between DM and CM

Risk of Bias Within Studies

Randomised controlled trials (RoB 2): Four randomised trial studies contributed PAR‑based occlusal outcomes: two comparing commercial clear aligners with fixed appliances; one comparing in‑house clear aligners with fixed appliances in complex extraction cases; and one comparing dental monitoring with conventional monitoring within clear‑aligner therapy, which was a retrospective secondary analysis of an RCT. All four trials were judged to have an overall risk of bias of “some concerns”. The randomisation process (D1) was generally adequate, but all trials were rated as “some concerns” regarding deviations from intended interventions (D2). While both participants and treating clinicians were not blinded to appliance type or monitoring strategy, lack of blinding alone does not establish bias in this domain. In addition, there were variations in the reporting of treatment adherence and co‑interventions. The study by Jaber et al. (2022) [27] was rated low risk for missing outcome data (D3), as no participants were lost, and complete PAR data were available. Gaikwad et al. (2025) [24] and Marks et al. (2024) [29] reported that a small proportion of randomised participants were lost to follow‑up. Gaikwad et al. [24] mentioned an intention-to-treat analysis with multiple imputations but reported results for 112 of 120 randomised participants. Marks et al. (2024) [29] reported discontinuations due to inconvenience or poor compliance and analysed patients with available pretreatment and post-treatment scans. Although this attrition was modest and similar across groups, its unclear effects on the PAR results led to “some concerns”. Alam et al. (2024) [23] reported complete follow‑up and was judged low risk for D3. Measurement of the outcome (D4) was rated as low risk in the existing assessment. PAR assessments that were blinded, calibrated, and of high reliability were documented in studies by Gaikwad et al. (2025) [24], Jaber et al. (2022) [27], and Marks et al. (2024) [29]. Alam et al. (2024) [23] did not report equivalent details, thus limiting support for its low-risk judgement. While the study by Jaber et al. (2022) [27] was rated as low risk for selection of the reported result (D5), the trial was retrospectively registered. As it was not established that the outcomes and analyses were specified before results were available, the low-risk judgement needs to be supported by evidence confirming prespecification. For Gaikwad et al. (2025) [24], Alam et al. (2024) [23], and Marks et al. (2024) [29], the absence of a clearly registered protocol for the specific PAR analysis and limited detail on pre‑specified secondary outcomes led to “some concerns” regarding selective emphasis. As shown in Figure 2, the RevMan traffic‑light plot shows “some concerns” in deviations from intended interventions and selective reporting across RCTs, with no trial at unequivocally low overall risk of bias. Figure 2 summarises the RoB 2 assessments of the four randomised trial studies.

**Figure 2:**
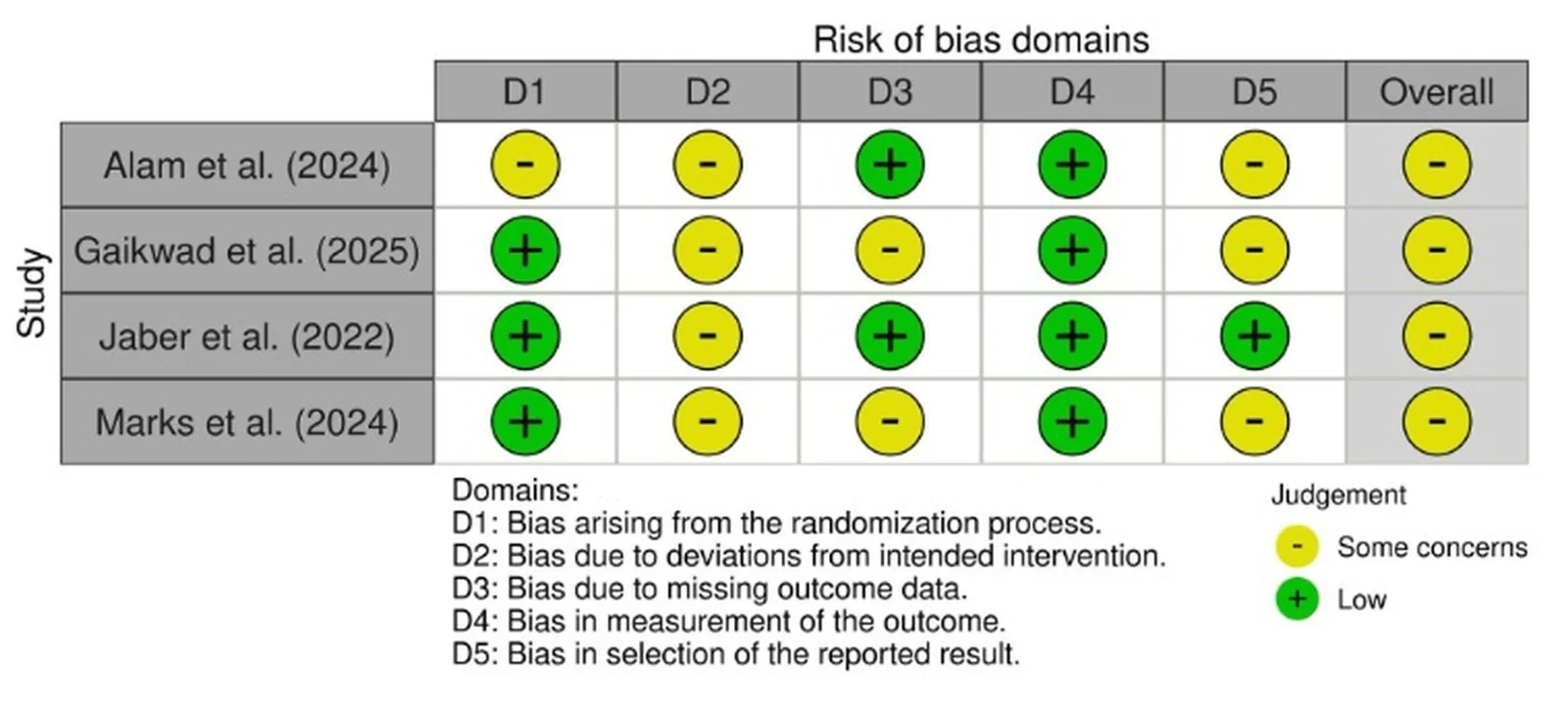
Risk of Bias in Randomised Controlled Trials (RoB 2 Traffic‑Light Plot) Studies assessed: Alam et al. (2024) [23], Gaikwad et al. (2025) [24], Jaber et al. (2022) [27], and Marks et al. (2024) [29]. The risk of bias in randomised controlled trials was assessed with the revised Cochrane RoB 2 tool. Colours indicate the risk as follows: low risk (green), some concerns (yellow), and high risk (red).

Non‑randomised studies (ROBINS‑I): Additional PAR-based data were obtained from three non‑randomised clinical studies, comprising two retrospective comparisons of clear aligners and fixed appliances [26,28], and one prospective clear‑aligner cohort with pre‑/post‑ and follow‑up PAR measurements [25].

Gu et al. (2017) [26] (retrospective case-control, Invisalign vs. fixed appliances, non‑extraction mild-moderate malocclusion) was judged to have a serious overall risk of bias. Treatment allocation was based on clinical decisions and patient preference, and only completed cases with full records were included, which may favour better outcomes in both groups. Lanteri et al. (2018) [28] (retrospective cohort, Invisalign vs. fixed appliances in anterior crowding with two‑year follow‑up) also had a serious overall risk of bias. Despite matching on age, sex, and baseline crowding and PAR, residual confounding is likely, and study inclusion was limited to those who have completed treatments from an experienced provider with available records. Follow‑up attrition was substantial; even though some losses and retention failures were explained, their potential effects on follow‑up outcomes were not formally accounted for, leading to at least moderate concern for missing data. Graf et al. (2021) [25] (prospective clear‑aligner cohort, effectiveness and stability using PAR) was rated at moderate overall risk of bias. As a single‑arm study, confounding by case selection and prognostic factors cannot be excluded, and recruitment may have favoured patients more suitable for aligners. However, within‑group pre‑/post‑comparisons do not ameliorate these concerns or enable comparison against the effectiveness of fixed appliances. A ROBINS‑I traffic‑light plot was generated in robvis, as shown in Figure 3. It highlights serious concerns for confounding and selection in both retrospective comparison studies and an overall rating of moderate risk for the prospective aligner cohort, with consistently low risk for outcome measurements across all three. Figure 3 summarises the ROBINS-I risk-of-bias assessments of the three non-randomised studies.

**Figure 3:**
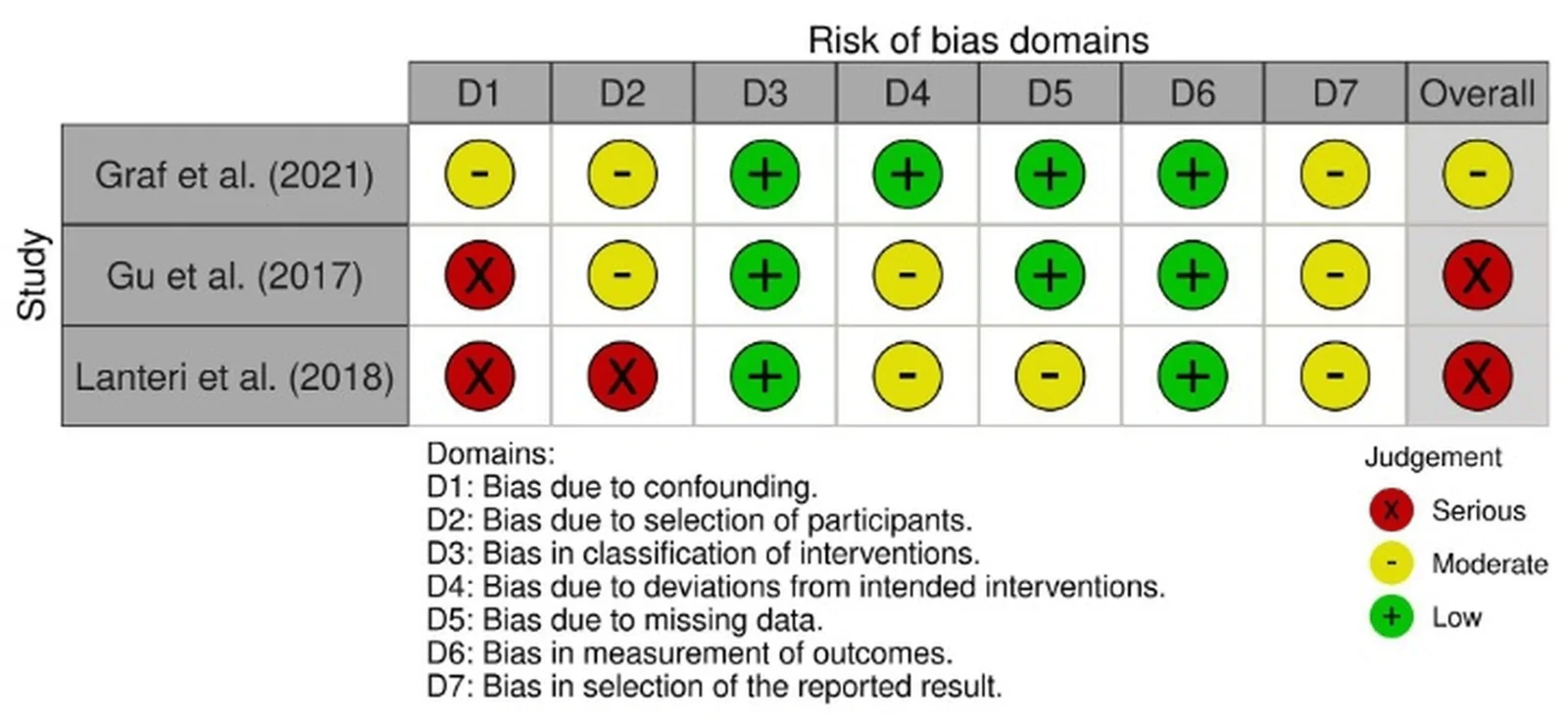
Risk of Bias in Non‑randomised Studies (ROBINS‑I Traffic‑Light Plot) Studies assessed: Graf et al. (2021) [25], Gu et al. (2017) [26], and Lanteri et al. (2018) [28]. A traffic‑light plot for the three non‑randomised clinical studies was generated using the ROBINS‑I tool and visualised with robvis. Colours indicate the risk as follows: low risk (green), moderate risk (yellow), serious risk (red), and, if present, no information (grey).

Data Synthesis

Occlusal improvement (percentage PAR reduction): Three controlled trials (Alam et al. (2024) [23], Gaikwad et al. (2025) [24], and Jaber et al. (2022) [27]; 248 participants) reported percentage reduction in UK‑weighted PAR scores between baseline and time of assessment, which was 12 months in Alam et al. [23] and the end of active treatment in the other two trials. All studies showed large improvements in both arms with mean PAR reductions of ≥75%. Additionally, fixed appliances achieved slightly higher mean reductions in two adult trials involving mild to moderate malocclusions (Alam et al. (2024) [23] and Gaikwad et al. (2025) [24]), and clear aligners produced slightly higher reductions in the extraction‑case trial done by Jaber et al. (2022) [27]. For Gaikwad et al. [24], recalculation from the published group means, standard deviations, and analysed sample sizes using a two-sided independent-samples t-test yielded p=0.01415, whereas the study reported p=0.082. The published summary data were retained in the meta-analysis. Random‑effects pooling demonstrated no statistically significant difference between clear aligners and fixed appliances (mean difference −2.17 percentage points; 95% CI −6.74 to 2.41; p=0.35; I²=73%; Figure 4). A sensitivity analysis excluding Alam et al. [23], whose 12-month PAR assessment did not confirm treatment completion, included the remaining two trials (148 participants) and showed no statistically significant difference (mean difference −0.51 percentage points; 95% CI −7.57 to 6.56; p=0.888). These findings indicate that no statistically significant difference in overall occlusal improvement was detected between clear aligners and fixed appliances when assessed using percentage PAR reduction, although heterogeneity between studies was substantial. Figure 4 presents the pooled comparison of percentage PAR reduction between clear aligners and fixed appliances.

**Figure 4:**
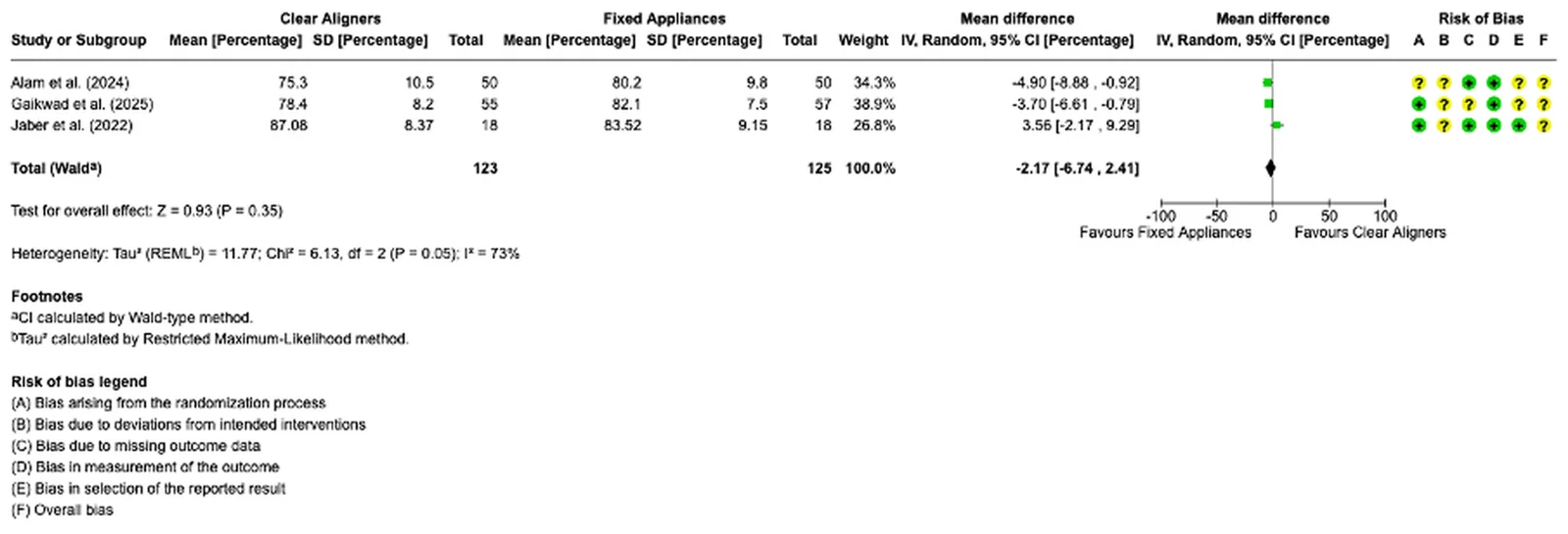
Forest Plot of Percentage Reduction in PAR at 12 Months or the End of Active Treatment for Clear Aligners vs. Fixed Appliances The figure illustrates a random-effects meta-analysis of percentage reduction in Peer Assessment Rating (PAR) scores from baseline to 12 months in the study by Alam et al. [23] and to the end of active treatment in studies by Gaikwad et al. [24] and Jaber et al. [27].

Proportion of patients achieving “great improvement” in PAR: Three studies (Gu et al. (2017) [26], Lanteri et al. (2018) [28], and Jaber et al. (2022) [27]; 332 participants) evaluated study-defined “great improvement” in PAR. Jaber et al. [27] used a reduction of at least 22 PAR points. Gu et al. [26] additionally included patients with baseline PAR below 22 who achieved a final PAR score of zero. Lanteri et al. [28] reported a percentage-based criterion (≥70%) in the Methods, with inconsistent wording in the table footnotes. The pooled analysis used each study’s reported classification. Clear aligners had event rates of 22.9-77.8% and fixed appliances had event rates of 45.8-83.3%; percentages were calculated based on the reported event counts. As shown in Figure 5, the pooled random‑effects risk ratio (RR) favoured fixed appliances numerically but did not reach statistical significance (RR 0.86; 95% CI 0.69 to 1.06; p=0.15; I²=0%). A sensitivity analysis excluding that of Lanteri et al. [28] yielded RR 0.72 (95% CI 0.39 to 1.32; p=0.288). Based on this evidence, neither modality has a robust advantage in delivering excellent occlusal outcomes by PAR criteria. Figure 5 presents the pooled comparison of patients achieving “great improvement” in PAR with clear aligners versus fixed appliances.

**Figure 5:**
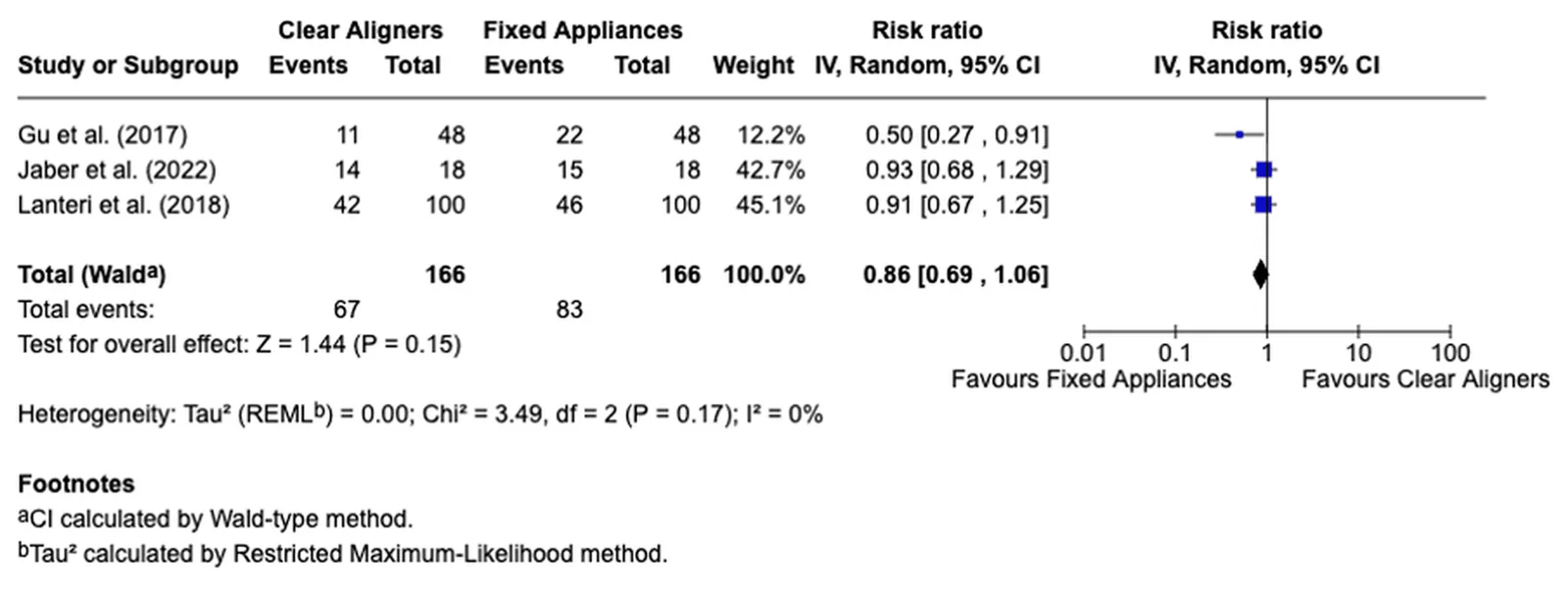
Forest Plot of “Great Improvement” in PAR for Clear Aligners vs. Fixed Appliances The figure demonstrates a random‑effects meta‑analysis of studies reporting the proportion of patients achieving great PAR improvement (≥22‑point reduction and/or final PAR 0). The effect measure is risk ratio (RR); RR >1 favours clear aligners, while RR <1 favours fixed appliances. Studies included: Gu et al. (2017) [26], Jaber et al. (2022) [27], and Lanteri et al. (2018) [28].

Treatment Duration and Efficiency

Four studies (Alam et al. (2024) [23], Gaikwad et al. (2025) [24], Gu et al. (2017) [26], and Jaber et al. (2022) [27]; 344 participants) reported total treatment time from appliance placement or first aligner to debond or delivery of retainers. Treatment duration estimates varied widely across studies and case‑mixes encompassing mild to moderate adult samples, which included both extraction and non‑extraction cases. Compared to fixed appliances, clear aligners had a modestly shorter treatment duration in the RCT done by Alam et al. (2024) [23], a longer treatment duration in the study done by Gaikwad et al. (2025) [24], and substantially shorter in the retrospective study done by Gu et al. (2017) [26]. In the severe extraction trial done by Jaber et al. (2022) [27], treatment duration with clear aligners was on average 2.9 months shorter, but the between‑group difference was not statistically significant.

Random‑effects pooling showed no statistically significant overall difference in treatment duration between clear aligners and fixed appliances (mean difference −1.48 months; 95% CI −5.45 to 2.49; p=0.46). As shown in Figure 6, there was very high heterogeneity (I²=96%), likely due to variations in malocclusion severity (extraction vs. non‑extraction), aligner type (Invisalign vs. in‑house), and study design (RCT vs. retrospective cohort). Taken together, these findings suggest that treatment time is highly context‑dependent, and current evidence across the included studies does not support a consistent advantage of either modality in treatment duration. Figure 6 presents the pooled comparison of treatment duration between clear aligners and fixed appliances.

**Figure 6:**
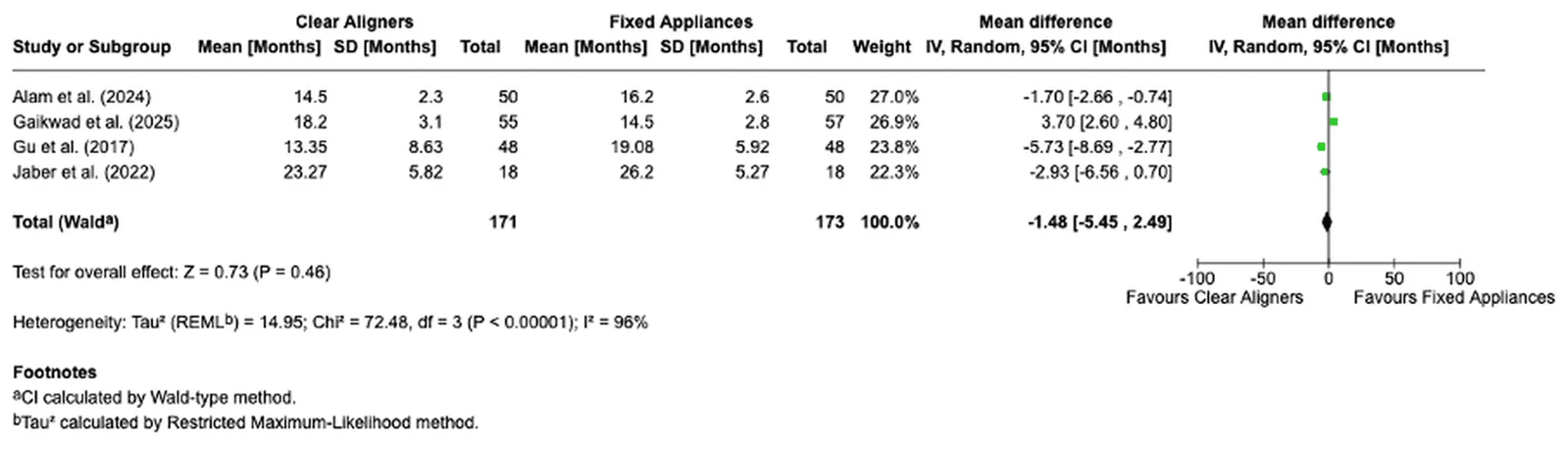
Forest Plot of Treatment Duration (months) for Clear Aligners vs. Fixed Appliances The figure shows a random‑effects meta‑analysis comparing total treatment time from appliance placement or first aligner to de-bond or retainer delivery. Mean differences (MD) <0 indicate shorter treatment duration with clear aligners, while MD >0 indicate shorter duration with fixed appliances. Studies included: Alam et al. (2024) [23], Gaikwad et al. (2025) [24], Gu et al. (2017) [26], and Jaber et al. (2022) [27].

Qualitative Synthesis of Component‑Level PAR Outcomes

Across the two PAR‑based comparative studies, clear aligners and fixed appliances produced broadly similar improvements in several components of the weighted PAR index encompassing maxillary and mandibular anterior alignment, anteroposterior, transverse, and vertical posterior occlusal relationships, overjet, overbite, and midline. In the extraction‑based RCT by Jaber et al. (2022) [27], severe crowding cases treated with in‑house clear aligners or fixed appliances achieved overall similar scores for post‑treatment posterior occlusion. Although a slight increase (<1 point) in the anteroposterior component was noted for the aligner group, mean differences between appliances for all three posterior components remained well below one PAR point (not statistically significant). Overjet and overbite components demonstrated substantial correction with both interventions, with the weighted overjet contributing the greatest share of total PAR reduction and overbite changes being more modest yet comparable between groups. Midline discrepancies also decreased similarly in both arms. The overall weighted PAR reduction exceeded 25 points in each group; based on the reported counts, 14/18 or 77.8% of clear‑aligner patients and 15/18 or 83.3% of fixed‑appliance patients were classified as “greatly improved.”

In the non‑extraction cohort study by Gu et al. (2017) [26], pretreatment distributions of all eight PAR components were closely matched between Invisalign and fixed‑appliance patients, and post‑treatment component scores again did not differ significantly. All components had mean values of below 1, except for the anteroposterior component that scored 2.06 for Invisalign and 1.69 for fixed appliances. Despite similar absolute and percentage reductions across individual components, the odds of achieving “great improvement” in the overall malocclusion were lower in the Invisalign group than in the fixed‑appliance group. That said, this categorical variation does not act as a significant between‑group difference in any individual PAR component. Taken together, these two studies suggest that at the level of individual PAR components, clear aligners can achieve anterior alignment and three‑dimensional posterior occlusal correction comparable to fixed appliances in both extraction and non‑extraction settings. While the non-extraction cohort studied by Gu et al. (2017) [26] demonstrated reduced odds of achieving great improvement with Invisalign, this finding should not be extrapolated to complex extraction cases.

Overall Certainty of Evidence (GRADE)

While the main meta‑analysis includes RCTs and one retrospective cohort, the GRADE certainty for duration is based on RCTs only. As shown in Table 3, the GRADE assessments were done only for the following RCTs: Alam et al. (2024) [23], Gaikwad et al. (2025) [24], and Jaber et al. (2022) [27]. The study done by Gu et al. (2017) [26] was excluded from the GRADE assessment as it had a non‑randomised design. When judged using GRADE principles, the certainty of evidence was rated as moderate for occlusal improvement outcomes (PAR Score Reduction (%)) and low for treatment duration, primarily due to risk of bias, inconsistency, and imprecision across the available studies.

**Table 3:** GRADE Summary of Findings for Clear Aligners vs. Fixed Appliances *The risk in the intervention group (and its 95% confidence interval) is based on the assumed risk in the comparison group and the relative effect of the intervention (and its 95% CI). CI: confidence interval; MD: mean difference GRADE Working Group grades of evidence: High certainty: we are very confident that the true effect lies close to that of the estimate of the effect; moderate certainty: we are moderately confident in the effect estimate: the true effect is likely to be close to the estimate of the effect, but there is a possibility that it is substantially different; low certainty: our confidence in the effect estimate is limited: the true effect may be substantially different from the estimate of the effect; very low certainty: we have very little confidence in the effect estimate: the true effect is likely to be substantially different from the estimate of effect.

Outcomes	Anticipated absolute effects^*^ (95% CI)		Relative effect (95% CI)	№ of participants (studies)	Certainty of the evidence (GRADE)	Comments
	Risk with Fixed appliances	Risk with Clear Aligners				
PAR Score reduction (%)		MD 2.17 percentage lower (6.74 lower to 2.41 higher)	-	248 (3 RCTs)	⨁⨁⨁◯ Moderate	Across RCTs, weighted PAR reductions were large and clinically meaningful in both groups, with no or only small differences between clear aligners and fixed appliances for mild–moderate cases and selected complex extraction cases. Fixed appliances may achieve slightly higher PAR % improvement in some trials, but the absolute differences are small and of uncertain clinical importance.
Treatment duration (months)		MD 0.12 months lower (4.16 lower to 3.92 higher)	-	248 (3 RCTs)	⨁⨁◯◯ Low	Downgraded for risk of bias and serious inconsistency (I²=96%); no consistent time advantage for either modality

Discussion

This systematic review and meta‑analysis synthesised PAR‑based evidence on occlusal outcomes of clear aligner therapy compared with conventional fixed appliances. Across seven included studies, clear aligners produced large reductions in weighted PAR scores, and pooled estimates indicated a lack of statistically significant difference in overall occlusal improvement relative to fixed appliances. For clarity, the primary comparative evidence in this review derives from the following five studies that directly compared clear aligners with fixed appliances: Alam et al. (2024) [23], Gaikwad et al. (2025) [24], Jaber et al. (2022) [27], Gu et al. (2017) [26], and Lanteri et al. (2018) [28]. Two studies, Graf et al. (2021) [25] (single-arm prospective cohort) and Marks et al. (2024) [29] (monitoring comparison within CAT), were used to derive supportive findings on the overall effectiveness of and monitoring strategies for clear aligners, rather than a direct comparison with fixed appliances. According to the studies done by Alam et al. (2024) [23], Gaikwad et al. (2025) [24], and Jaber et al. (2022) [27], totalling 248 participants, the percentage PAR reduction from baseline to the point of assessment did not differ significantly between modalities. In addition, the proportions of patients achieving “great improvement” differed across studies (Gu et al. (2017) [26], Lanteri et al. (2018) [28], and Jaber et al. (2022) [27]; 332 participants), with no statistically significant pooled advantage for either appliance. In the extraction‑based RCT by Jaber et al. (2022) [27], in‑house clear aligners and fixed appliances delivered broadly similar component‑level PAR outcomes. Based on the reported data, great improvement was achieved by 14/18 or 77.8% of clear‑aligner patients and 15/18 or 83.3% of fixed‑appliance patients. While the source labels these counts as 88.9% and 91.7%, respectively, it appears to be an inconsistency, and count-derived percentages are presented here. Likewise, Gu et al. (2017) [26] reported no statistically significant differences between Invisalign and fixed appliances in post‑treatment component‑level PAR scores in non‑extraction cases. However, Gu et al. (2017) [26] also showed that the odds of achieving great improvement were lower with Invisalign than with fixed appliances. This nuance aligns with the direction, but not the magnitude, of our “great improvement” meta‑analysis, in which the RR favoured fixed appliances without reaching statistical significance. That said, the pooled result does not establish that fixed appliances are superior in producing great improvement. The results are generally consistent with prior systematic reviews of clear aligner therapy, which have concluded that aligners can deliver acceptable occlusal outcomes in mild‑to‑moderate malocclusions but remain less predictable for complex tooth movements such as significant root torque, extrusion, correction of large anteroposterior discrepancies, and refinement of posterior occlusal contacts (Papadimitriou et al., 2018) [30].

The meta‑analysis of treatment duration found no consistent time advantage for either modality, with a pooled mean difference of approximately −1.5 months and very high heterogeneity across studies. This pattern aligns with previous comparative trials, which have reported mixed findings regarding treatment length for clear aligners versus fixed appliances, with some cohorts experiencing longer aligner treatment and others reporting similar or slightly shorter durations (Ke et al. (2019) [31]). Earlier systematic reviews by Papadimitriou et al. (2018) [30], Papageorgiou et al. (2020) [32], and Robertson et al. (2020) [33] concluded that clear aligners are effective for mild‑to‑moderate cases but less predictable for complex movements and detailed finishing. Comparatively, our results reinforce a balanced interpretation: aligners and fixed appliances achieve similar average PAR improvements, despite lingering concerns about challenging movements and detailed finishing. Based on our pooled results, fixed appliances do not have an established advantage to achieve an outcome of “great improvement”. Taken together, these data indicate that efficiency is highly context‑dependent. Rather than being determined solely by appliance type, it is possibly influenced by several factors including malocclusion complexity, extraction protocols, aligner system, staging strategy, and clinician expertise.

Evidence on post‑treatment stability was limited but broadly reassuring. Two cohorts with PAR‑based follow‑up reported that low post‑treatment PAR scores were largely maintained over short‑ to medium‑term retention, with only minimal changes between T1 and T2 [25,28]. Here, stability is defined based on the retention protocols used in the included cohorts. It does not establish that long‑term stability is independent of appliance type. Given the small sample sizes, absence of randomised long‑term data, and variability in retention protocols, any conclusions about comparative stability between appliances must be considered tentative. Although this review focused on PAR‑based occlusal outcomes, the wider literature highlights additional domains that may influence clinical decision‑making. These considerations, alongside the lack of statistically significant pooled differences in overall occlusal improvement, suggest that appliance choice should be individualised, taking into account patient preferences, compliance expectations, biomechanical demands, and clinician experience.

Study Limitations

This review has several limitations. The review protocol was not prospectively registered. First, the number of eligible studies was small, and only a subset contributed data to each meta‑analysis, limiting the precision of pooled estimates and the ability to explore heterogeneity fully. Second, the included studies were heterogeneous in terms of malocclusion severity, extraction status, aligner systems, retention protocols and study design. Specifically, a mix of randomised trials and retrospective cohorts was included, and risk‑of‑bias assessments frequently identified concerns regarding allocation concealment, deviations from intended interventions, and selective reporting. Furthermore, the 12‑month PAR assessment in Alam et al. [23] was assumed to represent the end-of-treatment assessment, even though treatment completion at that point was not confirmed, and reported mean treatment durations exceeded 12 months. This assumption limits the pooled estimate from being used as a comparison of final occlusal outcomes. Third, although PAR is a validated index for assessing occlusal improvement [16], it does not capture all aspects of treatment quality (e.g., root paralleling, detailed contact relationships, facial aesthetics or patient‑reported outcomes). Several potentially relevant clinical studies were excluded because they reported only other indices such as ABO‑OGS or purely dimensional measures. Fourth, reporting of variance measures was incomplete in some studies, necessitating reliance on percentage reduction or pre‑/post‑means without change‑score standard deviations, and precluding inclusion of certain datasets in specific analyses. Several inconsistences in the sources also affected interpretation. In the study by Gaikwad et al. [24], the reported between-group p-value for percentage PAR reduction did not agree with recalculation using the published means, standard deviations, sample sizes, and stated test. However, the published summary data were used in the meta-analysis. Finally, stability data were sparse, short‑ to medium‑term only, and derived exclusively from non‑randomised cohorts, resulting in very low certainty of evidence for this outcome.

Research Priorities and Future Directions

Future research should prioritise adequately powered RCTs that directly compare contemporary clear aligner systems with modern fixed appliance protocols in clearly defined malocclusion strata, including both non‑extraction and extraction cases. Trials should use standardised, weighted PAR and, ideally, additional indices to capture both occlusal quality and fine‑detail finishing, with blinded outcome assessment and prespecified analysis plans. In addition, longer‑term follow‑up with harmonised retention regimens is required to evaluate true post‑treatment stability and relapse patterns after aligner and fixed‑appliance therapy. Further studies should also address movement‑specific predictability and efficiency. For example, digital superimposition can be integrated with clinical indices. Studies should also incorporate patient‑reported outcomes and economic evaluations to contextualise occlusal findings within broader patient‑centred and health‑system perspectives.

## Conclusions

Clear aligners and fixed appliances showed no statistically significant differences in overall PAR improvement or treatment duration. These findings do not establish equivalence between the two treatments. Findings were also broadly similar across individual PAR components. However, the small number of eligible studies and substantial heterogeneity limit confidence in these findings. Larger, well-designed studies are needed to assess movement-specific predictability and treatment efficiency using digital superimposition alongside clinical indices. Patient-reported outcomes and economic evaluations would provide a fuller understanding of treatment effectiveness.
